# Correction: Synthesis, antibacterial activity, and mechanistic characterization of novel 9-substituted berberine derivatives

**DOI:** 10.1039/d6md90033j

**Published:** 2026-09-30

**Authors:** Jiaying Tong, Huamao Yang, Yeying He, Kejie Chen, Chaoyue Chen, Haoyi Han, Chenping Li

**Affiliations:** a Department of Pulmonary and Critical Care Medicine (PCCM), The First Affiliated Hospital of Wenzhou Medical University 1210 University Town Wenzhou 325000 Zhejiang China 15869612698@163.com; b Chemical Biology Research Center at School of Pharmaceutical Sciences, Wenzhou Medical University Wenzhou 325035 Zhejiang China; c Department of Pharmacy, Jiangmen Central Hospital Jiangmen 529030 Guangdong China

## Abstract

Correction for ‘Synthesis, antibacterial activity, and mechanistic characterization of novel 9-substituted berberine derivatives’ by Jiaying Tong *et al.*, *RSC Med. Chem.*, 2026, **17**, 951–964, https://doi.org/10.1039/d5md00825e.

The authors regret an error in the labelling of the panels in Fig. 3. The corrected version of Fig. 3 and the corresponding figure caption are shown here.

**Fig. 1:**
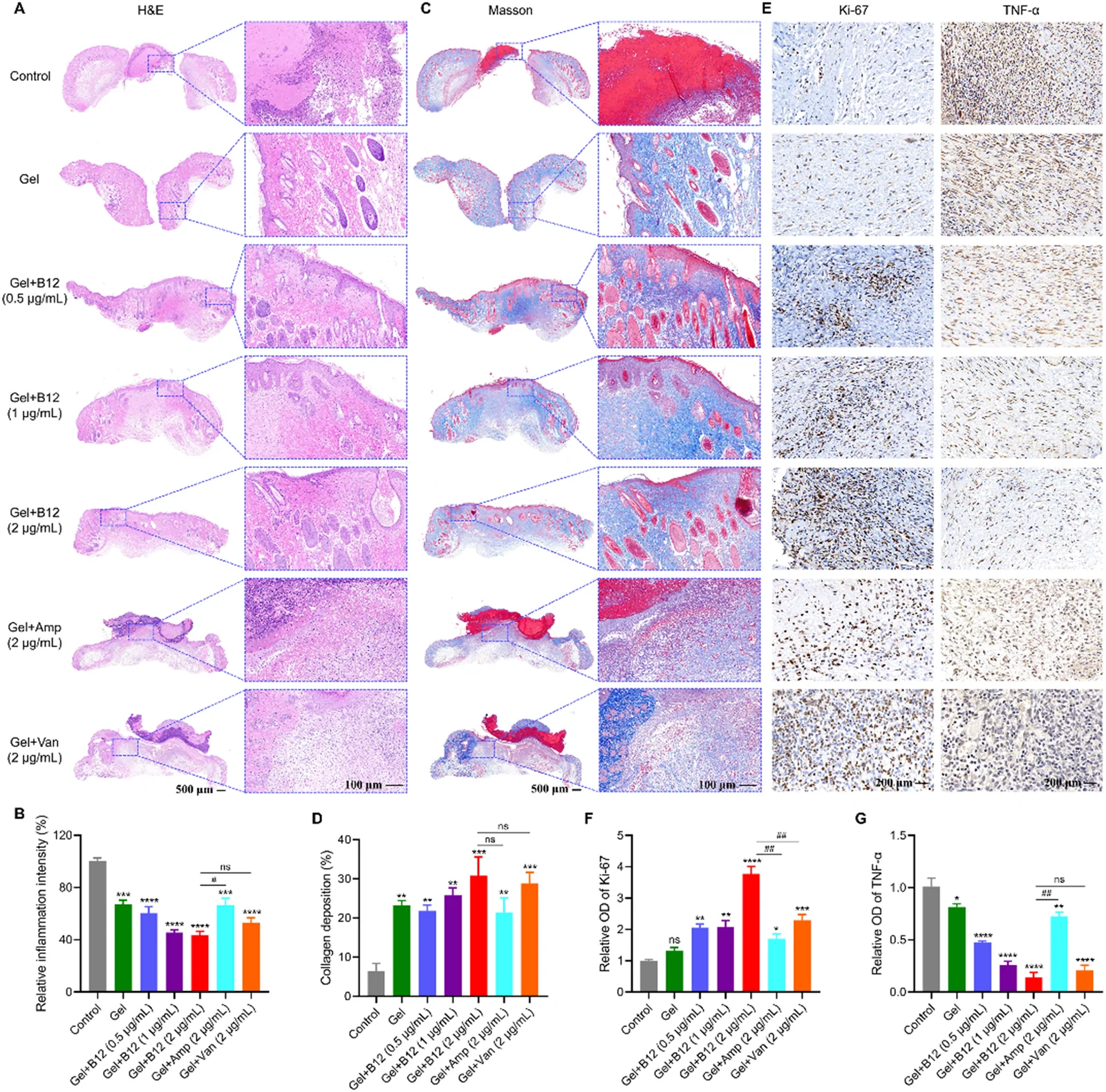
**B12** enhances cell proliferation and collagen deposition while reducing inflammation in infected wounds in a dose-dependent manner. (A) H&E staining of infected wounds in mice on the 11th day. (B) Statistical analysis of relative inflammation intensity from (A). (C) Masson staining of infected wounds in mice on the 11th day. (D) Statistical analysis of collagen deposition percentage from (C). (E) Immunohistochemistry of Ki-67 and TNF-α in infected wounds on the 11th day. (F) Statistical analysis of Ki-67 intensity from (E). (G) Statistical analysis of TNF-α intensity from (E). Error bars, mean ± SD. Ns, not significant; *p < 0.05; **p < 0.01; ***p < 0.001; ****p < 0.0001.

The Royal Society of Chemistry apologises for these errors and any consequent inconvenience to authors and readers.

